# Dr. Orrin A. Tompkins

**Published:** 1907-07

**Authors:** 


Dr. Orrin A. Tompkins, of Randolph, N. Y., died at his home June 11, 1907, aged 66 years. He was a graduate of the University of Buffalo, medical department, class of 1865, and for many years held an enviable reputation as a prominent practitioner of medicine in the community in which he lived.

